# A Decade of Leadership and Impact: Celebrating 10 Years of the ORS Spine Section

**DOI:** 10.1002/jsp2.70180

**Published:** 2026-04-13

**Authors:** Neharika Bhadouria, Rahul Gawri, Gabriela Graziani, Dennis E. Anderson, Ana V. Chee, Chitra L. Dahia, Ashish Diwan, Morgan B. Giers, Svenja Illien‐Junger, Anthony Kirilusha, David J. Nuckley, Derek H. Rosenzweig, Cheryle Séguin, Dmitriy Sheyn, Graciosa Q. Teixeira, Mauro Alini, Keita Ito, Robert L. Mauck, Daisuke Sakai, Jeannie Bailey, Aaron J. Fields, Sibylle Grad, Sarah E. Gullbrand, Nilsson Holguin, Rita Kandel, Zhen Li, Joshua Li, John T. Martin, Fackson Mwale, Grace D. O'Connell, Devina Purmessur, Nam Vo, Uruj Zehra, Nadeen O. Chahine, Lisbet Haglund, Judith A. Hoyland, Christine L. Le Maitre, Jeffrey C. Lotz, Makarand V. Risbud, Lachlan J. Smith, Simon Tang, James C. Iatridis, Karin Wuertz‐Kozak, Dino Samartzis

**Affiliations:** ^1^ Department of Orthopedics Icahn School of Medicine at Mount Sinai New York New York USA; ^2^ Schroeder Arthritis Institute, University Health Network (UHN) University of Toronto Toronto Ontario Canada; ^3^ Division of Orthopaedic Surgery, Department of Surgery McGill University Montreal Montreal Québec Canada; ^4^ Human Sciences and Life Quality Promotion Department University Rome San Raffaele Rome Italy; ^5^ Department of Orthopaedic Surgery Beth Israel Deaconess Medical Center & Harvard Medical School Boston Massachusetts USA; ^6^ Department of Orthopedic Surgery Rush University Medical Center Chicago Illinois USA; ^7^ Orthopedic Soft Tissue Research Program Hospital for Special Surgery New York New York USA; ^8^ Department of Cell & Developmental Biology Weill Cornell Medical College New York New York USA; ^9^ Department of Orthopaedic Surgery University of New South Wales Kogarah New South Wales Australia; ^10^ School of Chemical, Biological and Environmental Engineering Oregon State University Corvallis Oregon USA; ^11^ Department of Orthopaedics Emory School of Medicine & Georgia Institute of Technology Atlanta Georgia USA; ^12^ Dynanet Corporation Elkridge Maryland USA; ^13^ VB Spine New York New York USA; ^14^ Department of Orthopedic Surgery University of Connecticut Farmington Connecticut USA; ^15^ Department of Physiology and Pharmacology, Schulich School of Medicine and Dentistry Western University London Ontario Canada; ^16^ Regenerative Medicine Institute, Department of Orthopaedics Cedars‐Sinai Health Sciences University Los Angeles California USA; ^17^ Institute of Orthopaedic Research and Biomechanics, Center for Trauma Research Ulm Ulm University Medical Center Ulm Germany; ^18^ AO Research Institute Davos Platz Switzerland; ^19^ Department of Biomedical Engineering Eindhoven University of Technology Eindhoven the Netherlands; ^20^ Department of Orthopaedic Surgery University of Pennsylvania Philadelphia Pennsylvania USA; ^21^ Translational Musculoskeletal Research Center Corporal Michael J. Crescenz VA Medical Center Philadelphia Pennsylvania USA; ^22^ Department of Orthopaedic Surgery Tokai University School of Medicine Isehara Kanagawa Japan; ^23^ Department of Orthopaedic Surgery University of California San Francisco San Francisco California USA; ^24^ Pathology and Laboratory Medicine, Mt Sinai Hospital and Laboratory Medicine and Pathobiology, Temerty Faculty of Medicine University of Toronto Toronto Ontario Canada; ^25^ Department of Orthopaedic Surgery University of Virginia School of Medicine Charlottesville Virginia USA; ^26^ Department of Mechanical Engineering University of California, Berkeley Berkeley California USA; ^27^ Department of Biomedical Engineering The Ohio State University Columbus Ohio USA; ^28^ Department of Orthopaedic Surgery University of Pittsburgh Pittsburgh Pennsylvania USA; ^29^ Department of Anatomy University of Health Sciences Lahore Pakistan; ^30^ Department of Orthopedic Surgery Columbia University New York New York USA; ^31^ Department of Biomedical Engineering Columbia University New York New York USA; ^32^ Shriners Hospital for Children McGill University Montreal Québec Canada; ^33^ Division of Cell Matrix Biology and Regenerative Medicine, School of Biological Sciences The University of Manchester Manchester UK; ^34^ School of Medicine and Population Health The University of Sheffield Sheffield UK; ^35^ Cell Biology and Regenerative Medicine Thomas Jefferson University Philadelphia Pennsylvania USA; ^36^ Department of Orthopedics Washington University in Saint Louis St. Louis Missouri USA; ^37^ Department of Biomedical Engineering Rochester Institute of Technology Rochester New York USA

**Keywords:** consortium, degeneration, disc, low back pain, ORS, Orthopaedic Research Society, spine

As we celebrate the 10th anniversary of the Spine Section of the Orthopaedic Research Society (ORS), we reflect on a decade defined by visionary leadership, scientific excellence, and a steadfast commitment to advancing spine research. What began as a focused initiative within the ORS to unify spine investigators has grown into a vibrant, international community of scientists and clinicians at all career stages dedicated to transforming musculoskeletal health through collaboration, education, discovery, and translation. Since its inception, the ORS Spine Section has expanded not only in membership but also in scientific scope, global engagement, and leadership development. This growth is a testament to the dedication of past and present Chairs and Officers, whose selfless contributions and countless hours of service cemented the foundation on which the Section proudly stands today.

## 1 Founding Vision and Early Momentum

**FIGURE 1:**
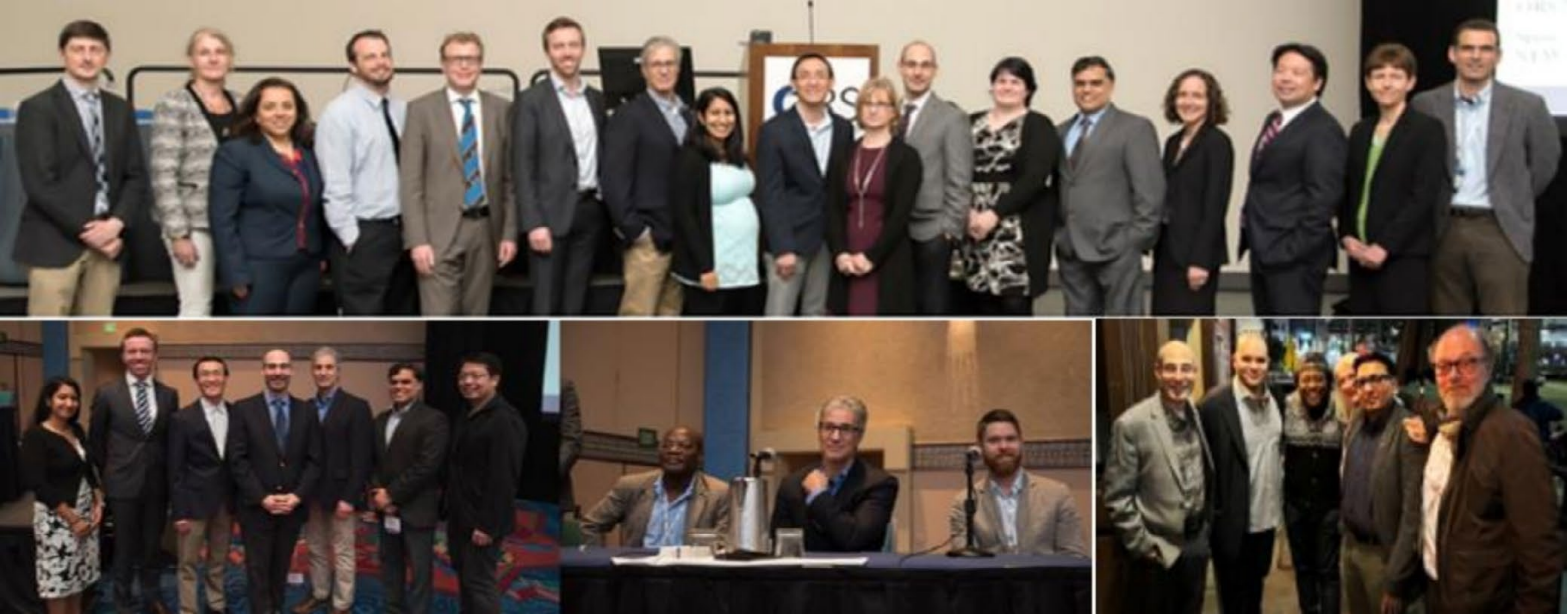
Initial members of the ORS Spine Research Interest Group (RIG) and ORS Spine Section members. Top Row (left to right): Lachlan J. Smith, Lisbet Haglund, Nadeen O. Chahine, John T. Martin, Benjamin Gantenbein, Casper Bindzus Foldager, Jeffrey C. Lotz, Devina Purmessur, Nam Vo, Judith A. Hoyland, James C. Iatridis, Christine L. Le Maitre, Makarand V. Risbud, Sarah E. Gullbrand, Daisuke Sakai, Sibylle Grad, Aaron J. Fields. Bottom Row: (far left) Devina Purmessur, Casper Bindzus Foldager, Nam Vo, James C. Iatridis, Jeffrey C. Lotz, Makarand V. Risbud, Daisuke Sakai; (center) Fackson Mwale, Jeffrey C. Lotz, Robby Bowles (far right) James C. Iatridis, Dino Samartzis, Rahul Gawri, Mauro Alini.

The first ORS Spine Research Interest Group (RIG) was organized in 2011 by its founding organizers: Fackson Mwale, Daisuke Sakai, Makarand V. Risbud, Rita Kandel, and Sibylle Grad. Their vision was to create a collaborative platform for researchers dedicated to advancing spine science within the ORS. Following the initial success of the RIG, leadership and participation steadily expanded over the subsequent years with the support and contributions of many members of the spine research community. Among those who played important roles in its early growth were James C. Iatridis, Mauro Alini, Lisbet Haglund, Christine L. Le Maitre, Nam Vo, Devina Purmessur, Judith A. Hoyland, and Jeffrey C. Lotz, along with many other dedicated researchers and ORS staff, such as Bailey McMurray and others who contributed to the development and success of the group (Figure 1).

The RIG played a pivotal role in building a collaborative network and achieving early scientific milestones, including a consensus article on nucleus pulposus cell phenotypic markers [1], a *Journal of Orthopaedic Research* “virtual issue” highlighting key spine publications [2, 3], and a review on spinal aging [4]. Building on this momentum, the Section was formally established in 2016 with James C. Iatridis serving as its first Chair (2016–2017). During this formative period, the leadership clarified the Section's mission, launched its website and newsletter, initiated webinars, introduced social events at the ORS Annual Meeting and implemented member surveys to guide priorities. These foundational efforts, anchored in collaboration, mentorship, and scientific excellence, helped establish a vibrant and inclusive spine research community. From its inception, the Section embraced broad scientific integration, bringing together expertise in intervertebral disc biology, pain, spine biomechanics, mechanobiology, tissue engineering, biomaterials, inflammation research, computational modeling, imaging sciences, and clinical translation. This interdisciplinary approach became a defining characteristic of the Section and remains central to its continued success.

## 2 Growth of ORS Spine Section Membership

**FIGURE 2:**
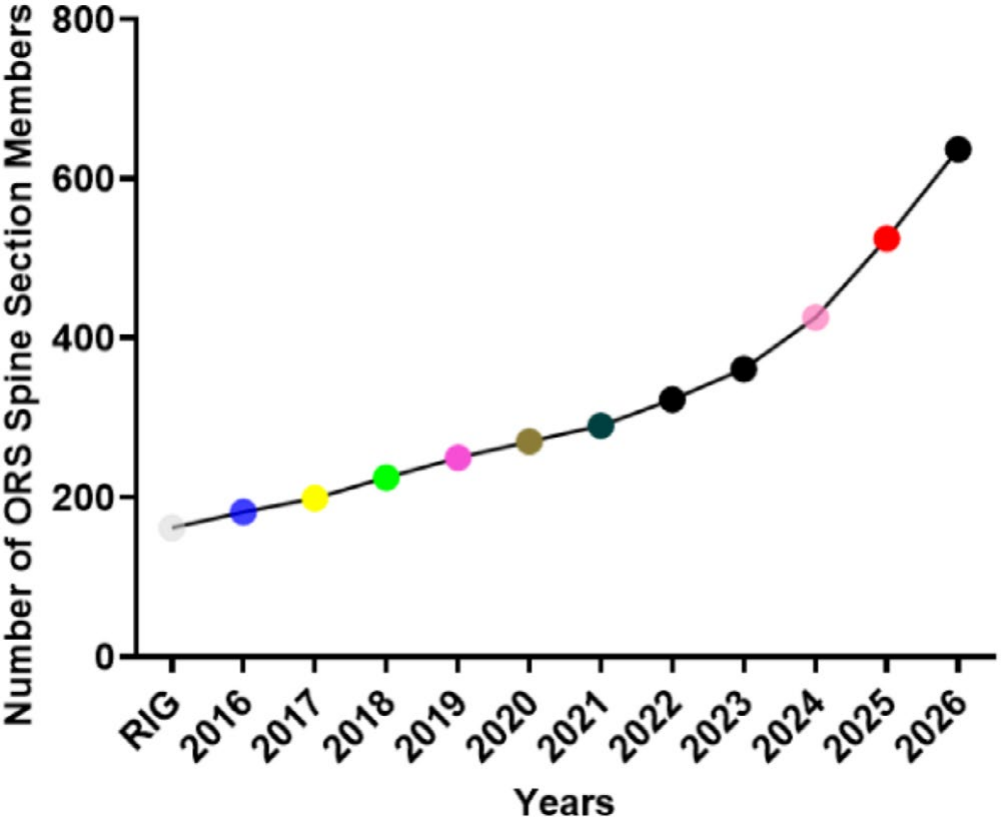
ORS Spine Section membership numbers stratified by year.

Over time, the ORS Spine Section evolved into a vibrant community hosting Section Symposia and social events that helped break down traditional barriers between faculty and trainees. These gatherings created a welcoming space where ideas were exchanged freely, and mentorship naturally developed, helping to nurture the next generation of spine research leaders. Furthermore, the growth of the Section throughout the years has seen an exponential growth in membership and engagement. For example, in 2015, the RIG had 162 members; whereas by March of 2026 the membership increased to 637 (Figure 2), an extraordinary 293% increase. In short, the ORS Spine Section has transformed from a small circle of specialists into a thriving international network. Today, it serves as a global nexus of multidisciplinary experts who come together to collaborate, share knowledge and drive the future of spine research and innovative clinical impact.

## 3 Leadership Through the Years

**FIGURE 3:**
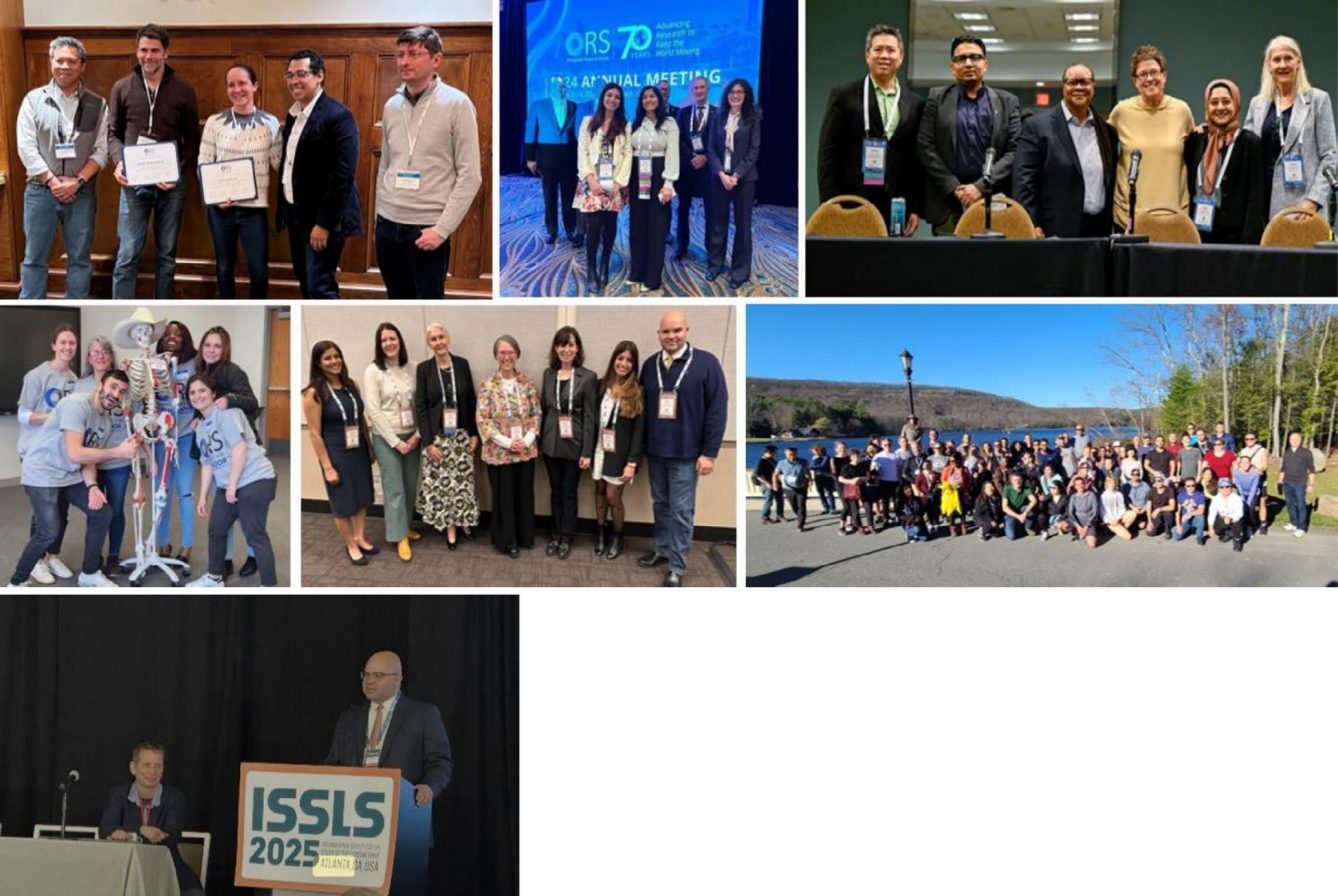
Highlights from ORS Spine Section Initiatives at ORS Annual and Co‐Sponsored Meetings: Top Row: (left) Award Recognitions: Simon Tang, Derek H. Rosenzweig, Sarah E. Gullbrand, Nilsson Holguin, Lachlan J. Smith; (center) “Thinking Outside the Box: Exploring Innovative Solutions to Unmet Clinical Needs in Spine” Symposium, co‐organized with the North American Spine Society (Guest Society), ORS Spine Section, ORS Innovation Section and ORS Industry Alliance Committee: (Back Row, left to right) Dino Samartzis, Rowley Hazard, Ben Goss, Mark Erwin, Zorica Buser, (Front Row, left to right) Gabriela Graziani, Neharika Bhadouria; (right) Spine Section Symposium Workshop and Panel discussion: Simon Tang, Rahul Gawri, Kelly Cross (workshop guest), Molly Grisham (workshop guest), Hagar. M. Kenawy, Lisbet Haglund; Center Row: (left) Spine Section members participating in ORS (Open Door Initiative) activities (Sade Clayton, Christopher Panebianco, Remy E Walk, Kaitlyn Broz); (center) “Exploring Unmet Needs in Women Empowerment: Integrating Gender Diversity as a Factor and Advancing Women in the Orthopaedic Field” Symposium in collaboration with the ORS Spine Section, ORS Women Leadership Forum and the Pediatric Orthopaedic Society of North America (Guest Society): (left to right) Neharika Bhadouria, Sarah Greising, Lisbet Haglund, Michelle Caird, Leesa M. Galatz, Gabriela Graziani, Dino Samartzis and (right) Group networking activities (post‐hike group photo) at ORS/PSRS meeting at Skytop, Pennsylvania, USA; Bottom Row: Spine Section members, Svenja Illien‐Junger and Dino Samartzis Co‐Chairing the ISSLS‐ORS Sports and Spine Symposium at the 2025 ISSLS Annual Meeting.

The growth and impact of the ORS Spine Section are closely intertwined with the vision, leadership, and sustained efforts of its past Chairs. Each Chair brought distinctive strengths and priorities that not only advanced the section during their respective terms but also contributed to a broader foundation for its continued evolution and success. Besides the Chair, the Section maintains a strong organizational structure that includes roles of Past Chair, Chair‐Elect, Secretary, Members‐at‐Large, and Officers responsible for education, research, membership, funding agency liaison, industry liaison, clinical society liaison, translational research liaison, and a journal representative. These roles continue to evolve to meet emerging needs. For example, during the COVID‐19 pandemic, the Section established a COVID‐19 Task Force that aimed to understand the pandemic's impact on research and the research environment [5, 6] (Table 1). The ORS Spine Section also provides an excellent pathway for promoting Officers and, most importantly, for nurturing the next generation of Section research leaders. Collectively, the contributions of these Chairs and Officers reflect a sustained trajectory of growth, innovation, and community building that has positioned the Section as an increasingly visible and influential entity within the field. Details regarding the Chairs and Officers serving during their respective terms and additional key contributions are provided in Table 1, and photos reflecting the journey are displayed in Figure 3.

**TABLE 1:** ORS spine section chairs and section officers (2016–2026).

Past chairs	Term	Officers during term	Key contributions
James C. Iatridis	2016–2017	Chair‐Elect: Judith A. Hoyland; Secretary: Christine L. Le Maitre; Treasurer: Jeffrey C. Lotz; Research Chair: Makarand Risbud; Education Chair: Nam Vo; Membership Co‐Chairs: Devina Purmessur, Daisuke Sakai	Contributed to RIG and its transition to the Spine Section; established the Section's leadership structure, launched the Section's website and newsletter; introduced webinars and social events; and supported the integration of *JOR Spine*.
Judith A. Hoyland	2017–2018	Chair‐Elect: Makarand V. Risbud; Secretary: Christine L. Le Maitre; Treasurer: Jeffrey C. Lotz; Education Chair: Nadeen O. Chahine; Research Chair: Sibylle Grad; Membership Co‐Chairs: Devina Purmessur, Daisuke Sakai (* Information correct to the best of our knowledge.)	Strengthened the membership; partnered with the ISSLS; led 2018 Spine Symposium; organized session at eCM Conference “Cartilage & Disc”; introduced Trainee Poster Teaser Session.
Makarand Risbud	2018–2019	Chair‐Elect: Jeffrey C. Lotz; Secretary: Grace O'Connell; Treasurer: Christine L. Le Maitre; Research Chair: Sibylle Grad; Education Chair: Nadeen O. Chahine; Membership Co‐Chairs: Devina Purmessur, Aaron Fields; Member‐at‐Large: Lisbet Haglund, Lachlan J. Smith, Joshua Li, David Nuckley; Section Past Chair: Judith A. Hoyland	Continued to strengthen scientific programming and foster community engagement through organizing sessions at the ORS Annual Meeting and leading ORS/PSRS symposia.
Jeffrey C. Lotz	2019–2020	Chair‐Elect: Nadeen O. Chahine; Secretary: Grace O'Connell; Treasurer: Christine L. Le Maitre; Research Chair: Lisbet Haglund; Education Chair: Lachlan J. Smith; Membership Co‐Chairs: Aaron Fields, Sarah E. Gullbrand; Member‐at‐Large: Nilsson Holguin, Joshua Li, David Nuckley, Dino Samartzis; Section Past Chair: Makarand V. Risbud	Developed the ICORS workshop (ORS Spine/JOA/AO Foundation); led the JOR Spine/ORS Histopathology Initiative; introduced the US Pain Foundation to ORS; and proposed the ORS Spine Section Traveling Fellowship to the ORS Board of Directors.
Nadeen O. Chahine	2020–2021	Chair‐Elect: Christine L. Le Maitre; Secretary: Simon Tang; Treasurer: David Nuckley; Research Co‐Chairs: Lisbet Haglund, Grace O'Connell; Education Chair: Lachlan J. Smith; Membership Co‐Chairs: Sarah E. Gullbrand, John Martin; Member‐at‐Large: Nilsson Holguin, Aaron Fields, Zhen Li, Dino Samartzis; Section Past Chair: Jeffrey Lotz	Introduced SMART goals; established Peter Roughley Award and Travel Award; launched journal club series; initiated ORS Spine Section Virtual Research Series, collaborated with ORS Section leadership to analyze the Spine Section membership census across all ORS Sections, highlighting that the Spine Section has the highest membership among them.
Christine L. Le Maitre	2021–2022	Chair‐Elect: Lachlan J. Smith; Secretary: Simon Tang; Treasurer: David Nuckley; Research Co‐Chairs: Grace O'Connell, Dino Samartzis; Education Chair: Nilsson Holguin; Membership Co‐Chairs: John Martin, Uruj Zehra; Member‐at‐Large: Aaron Fields, Zhen Li, Morgan Giers, Cheryle Seguin; Covid19 Task Force Head: Lisbet Haglund; Funding Agency Liaison: Anthony Kirilusha; Section Past Chair: Nadeen O. Chahine	Established and led a COVID‐19 task force and the creation of the Section Funding Agency Liaison; initiated diversity awards and successfully secured NIH funding; expanded programming, including supporting the PSRS as a satellite meeting; directed initiatives focused on bullying and harassment awareness; achieved NIH support to broaden scientific meetings, evolving from a single spine session to a full afternoon of programming; organized session at eCM Conference “Cartilage & Disc”; contributed to consensus papers for the histology grading series and methodological improvement editions in *JOR Spine*; and spearheaded consensus publications on cell culture protocols for NP cells and NCs.
Lachlan J. Smith	2022–2023	Chair‐Elect: Lisbet Haglund; Secretary: David Nuckley; Treasurer: Zhen Li; Research Co‐Chairs: Simon Tang, Dino Samartzis; Education Chair: Nilsson Holguin; Membership Co‐Chairs: Rahul Gawri, Uruj Zehra; Members‐at‐Large: Morgan Giers, Cheryle Seguin, Jeannie Bailey; Covid19 Task Force Head: Lisbet Haglund; Funding Agency Liaison: Anthony Kirilusha; Past Chair: Christine Le Maitre	Secured a 3‐year NIH R13 grant; expanded the ORS Spine Section Symposium to a half‐day format featuring leadership, diversity, mentoring, and scientific sessions; supported trainee diversity travel awards; contributed to a *JOR Spine* perspective on spine research models; leadership Co‐Chaired the ORS/PSRS International Spine Research Symposium; and hosted a virtual workshop on NIAMS funding opportunities.
Lisbet Haglund	2023–2024	Chair‐Elect: Simon Tang; Secretary: David Nuckley; Treasurer: Zhen Li; Research Chair: Nilsson Holguin; Cheryle Seguin; Education Chair: Morgan Giers; Membership Co‐Chairs: Rahul Gawri, Jeannie Bailey; Members‐at‐Large: Svenja Illien‐Junger, Karin Wuertz‐Kozak and, Dennis Anderson, Funding Agency Liaison: Anthony Kirilusha; Clinical Society Liaison: Dino Samartzis; Past Chair: Lachlan J. Smith	Led scientific publications addressing bullying, harassment and discrimination; coordinated a symposium with NASS and ORS; continued to promote diversity, mentorship, and collaborative scientific efforts.
Simon Tang	2024–2025	Chair‐Elect: Dino Samartzis; Secretary: Rahul Gawri; Treasurer: David Nuckley; Research Chair: Nilsson Holguin; Cheryle Seguin; Education Co‐Chairs: Morgan Giers, Svenja Illien‐Junger; Membership Co‐Chairs: Dmitriy Sheyn, Jeannie Bailey; Members‐at‐Large: Karin Wuertz‐Kozak, Ana Chee, Dennis E. Anderson, Funding Agency Liaison: Anthony Kirilusha; Task Force (Grants): Lachlan J. Smith; Past Chair: Lisbet Haglund	Established a dedicated Awards Committee to oversee the growing portfolio of the Section's honors; member‐driven symposium survey was enhanced to guide future scientific programming; Section intern program was developed to engage trainees in Section leadership and cultivate future Officers; diversity and outreach initiatives were strengthened and included the collaboration with the Open Door program to recognize leadership in STEM engagement at ORS Annual Meetings; further facilitated the partnership with the ISSLS for joint symposiums.
Dino Samartzis	2025–2026	Chair‐Elect: Karin Wuertz‐Kozak; Secretary: Rahul Gawri; Treasurer: David Nuckley; Research Chair: Gabriela Graziani; Education Co‐Chair: Svenja Illien‐Junger, Ana Chee; Membership Co‐Chairs: Dmitriy Sheyn, Derek Rosenzweig; Members‐at‐Large: Chitra Dahia, Graciosa Teixeira; Translational Research Taskforce Liaison: Morgan Giers; Funding Agency Liaison: Laurel Kuxhaus; Clinical Society Liaison: Ashish Diwan; Industry Liaison: Anthony Kirilusha; *JOR Spine* Liaison: Cheryle Seguin; Past Chair: Simon Tang	Expanded the Section's multidisciplinary and global reach; established strong partnerships with international spine organizations, such as the EORS, ISSLS, NASS, Spine Society of Germany, and AO Spine, with co‐branded symposia programming and knowledge exchange; guided the Section's first international meeting as part of Spineweek 2027; organized and hosted webinars, such as the ORS‐wide event “Navigating the New Normal: Strategies for Success in a Shifting NIH Landscape”; increased membership; implemented initiatives to support young investigators; curation, celebration and programming of the Section's 10‐year anniversary milestone via publishing and in‐person events; creation of the *JOR Spine* and the Translational Research Liaison positions; established the new Philotimo Award; and strengthened the Section's visibility, governance, outreach and impact in the spine research community.

Abbreviations: eCM, european cells and material; EORS, European Orthopaedic Research Society; ICORS, International Combined Research Societies; ISSLS, International Society for the Study of the Lumbar Spine; NASS, North American Spine Society; NC, notochordal cells; NIAMS, National Institute of Arthritis and Musculoskeletal and Skin Diseases; NIH, National Institutes of Health; NP, nucleus pulposus cells; PSRS, Philadelphia Spine Research Symposium; RIG, Research Interest Group; SMART, specific, measurable, achievable, relevant and time‐bound; STEM, science, technology, engineering and mathematics.

In addition, within the ORS Spine Section, the Membership Task Force plays an essential role in coordinating activities throughout the year, including sessions at the ORS Annual Spine Symposium and related co‐sponsored meetings. Former Task Force members, including (but not limited to) Sade Williams Clayton, Jasmine Xiao, Zhirui Jiang, Daniele Zuncheddu, and Nina Shirley Tang, made important contributions to the Section's initiatives. Current members Neharika Bhadouria, Luca Ambrosio, Andres Bonilla, Giselle Kaneda, and Jordy Schol continue this work, advancing the Task Force's mission and helping shape the future of the Section.

## 4 Scientific and Community Impact

What began as a small, focused group has grown into a vibrant, global community of scientists and clinicians united by a shared passion for advancing spine science, fostering collaboration and mentoring the next generation of researchers. Together, the Section works toward a common mission: to advance spine research and develop solutions that relieve pain and restore quality of life. At the heart of this mission is a commitment to serve patients whose lives are disrupted by spine conditions. Across countries, cultures and disciplines, our community is bound by the responsibility to bring hope and sustained innovation, even in the face of global challenges (e.g., limited research funding). It is this shared vision and collaborative spirit that sustains the Section's passion and drives progress day by day. The ORS spine community continues to strengthen collaborations, advance research and inspire discoveries that bring hope to those in need.

The ORS Spine Section Symposium has become a centerpiece of the ORS Annual Meeting, fostering cross‐disciplinary dialogue and highlighting cutting‐edge spine research. Through collaborations with international societies, such as NASS, ISSLS, and others, the Section has expanded its global reach and created an inclusive platform that connects spine investigators worldwide. The Section also partners with other ORS sections/committees, including the Preclinical Models Section [7], the ORS Innovation Committee, the ORS Women Leadership Forum, and the ORS Industry Alliance Committee, to co‐organize joint sessions that encourage interdisciplinary exchange and collaboration.

Beyond scientific programming, the Spine Section has demonstrated a strong commitment to community support, collaboration, and professional, united culture. The continued meaningful discussions, debates and future perspective talks organized during the Spine Section Symposium at the ORS Annual Meeting and also virtual sessions organized throughout the year has forged multidisciplinary and multicentric teams after they found common research interests and complementary expertise, and has led to many successful grant applications, promoted trainee mobilization and enabled pursuing innovative high‐risk high‐reward research in the spine field. Many Section members are clinicians and/or clinician‐scientists, and through one‐on‐one interactions and mentorship, they have helped cultivate the development of clinical translational scientists while guiding fundamental science trainees to think critically and develop translational therapeutic solutions that leverage their training. In the era of fast‐paced analyses of large datasets impacting the ever‐changing research landscape, the Section has achieved its vision and mandate of bringing together multidisciplinary expertise on a common platform. Many trainees engaged with the section's scientific programming and networking events; as a result, they have rechanneled their career trajectories and pursued advanced training in fields that seemed at one stage unreachable.

The ORS Spine Section is also instrumental in supporting and helping the Philadelphia Spine Research Society's (PSRS) biannual international meeting. This conference is in the style of a “Gordon Conference,” and was established by Section members prior to the elevation of the Spine RIG to a full ORS Spine Section. The discussions on hot research topics during the Spine Section Scientific Symposium at the ORS Annual Meeting are followed up at the PSRS meeting via presentations and invited keynote talks, and a more comprehensive workshop session is organized at the forthcoming Section symposium.

The Spine Section supports its community not only through scientific collaboration, but also during extraordinary circumstances, helping sustain both community engagement and scientific progress. As previously mentioned, during the COVID‐19 pandemic, the Section established a COVID Task Force to help the research community navigate unprecedented challenges [5, 8, 9]. The Section has also played an active role in fostering a respectful and inclusive scientific environment by educating members on topical issues, such as bullying, harassment and discrimination [6], and by promoting awareness of best practices and codes of conduct in research through dedicated publications and discussions.

Scientific excellence and trainee engagement are further recognized through multiple awards and initiatives, including the Peter Roughley Mentorship Award (Table 2), ORS Podium presentations, ORS Diversity Awards, Poster Awards, Trainee Travel Fellowships (Table 3), active involvement of Section members in the Open Door program to educate school children about orthopedic research, the spine and how to pursue a career in the sciences, and periodic Spine Section newsletters (The Spinal Column, https://www.ors.org/research‐section‐newsletters/#cb1c86c48ccf85438) (Figure 3). Programs such as the Mentor‐Mentee Match Program and leadership development opportunities help cultivate and support the next generation of spine investigators. The Section also organizes forward‐looking symposia to address emerging challenges and opportunities in the field. These have included discussions on the evolving NIH funding landscape, addressing unmet clinical needs and initiatives focused on women's empowerment in spine research and leadership (Figure 3). By integrating groundbreaking science, mentorship, recognition and global collaboration, the ORS Spine Section continues to advance discovery, translate innovation into improved patient care and unite a worldwide community dedicated to improving spine health.

**TABLE 2:** Dr. Peter Roughley award recipients.

Year	Recipient	Home affiliation	Host/where worked with award	Mentor(s)/project focus
2026	Rachel Thompson	PhD candidate, Oregon State University (Mentor—Morgan Giers)	UCSF Department of Orthopaedic Surgery, San Francisco, CA, USA	Mentor: Jeannie Bailey/To identify distinct degenerative disc disease phenotypes using principal component & K‐means cluster analyses
2025	Emily Sharp	PhD candidate, University of Pennsylvania, USA (Mentors—Robert L. Mauck, Sarah E. Gullbrand)	AO Research Institute (ARI), Davos, Switzerland	Mentor: Sibylle Grad/To study efficacy of multifunctional biomaterials with an innovative six‐degree‐of‐freedom bioreactor
2024	Janitri Venkatachala Babu	PhD Candidate, Rochester Institute of Technology, USA (Mentor—Karin Wuertz‐Kozak)	University of Minnesota, Minneapolis, MN, USA	Mentor: Laura Stone/To research on TRPC6 ion channel's role in intervertebral disc degeneration
2023	Jennifer Gansau	Postdoctoral Fellow, Icahn School of Medicine at Mount Sinai, New York, USA (Mentor—James C. Iatridis)	AO Research Institute (ARI), Davos, Switzerland	Mentors: Mauro Alini, Tiziano Serra, Junxuan Ma/To connect IVDD mechanisms with DRG organ culture model
2021	Josette van Maanen	PhD student, Utrecht University, Utrecht, Netherlands (Mentor—Marianna A. Tryfonidou)	Sheffield Hallam University, Sheffield, UK	Mentor: Christine Le Maitre/To study notochordal extracellular vesicles in NP explants and learn intervertebral disc explant culture techniques

*Note:* Website: https://www.ors.org/ors‐dr‐peter‐roughley.

**TABLE 3:** ORS Spine Section travel fellowship recipients.

Year	Recipient	Home affiliation	Host/where worked with award	Mentor(s)/project focus
2025	Brianna Orozco	University of Pennsylvania, Philadelphia, PA, USA (Mentor—Sarah E. Gullbrand)	University of California San Francisco, San Francisco, CA, USA	Mentor: Aaron Fields/To conduct and replicate convection studies through the human endplate tissue samples which include both the cartilage and bony endplate
2024	Emma Coltoff	Wake Forest University School of Medicine, NC, USA (Mentor—Philip Jayson Brown)	McGill University, Montreal, QC, Canada	Mentor: Mark Driscoll/To translate a novel six‐degree‐of‐freedom spine biomechanical testing method into finite element simulations to better characterize spinal mechanics
2023	Jenna Wahbeh	Luskin Orthopaedic Institute for Children, LA, USA (Mentor—Sophia Sangiorgio)	University of Waterloo, Waterloo, Canada	Mentor: Stewart McLachlin/To validate the innovative 3D printed cervical vertebral body for biomechanical assessments of cervical devices
2022	Irina Heggli	University of Zurich, Switzerland (Mentor—Stefan Dudli)	University of California San Francisco, San Francisco, CA, USA	Mentor: Aaron Fields/To explore the effects of activated neutrophils on cartilage endplate integrity and biochemical composition.
2021	Neharika Bhadouria	Purdue University, West Lafayette, IN, USA/IUPUI, IN, USA (Mentor—Nilsson Holguin)	Washington University in Saint Louis, MO, USA	Mentor: Simon Tang/To investigate the effects of the FDA approved bone drug raloxifene on the biomechanical properties of the intervertebral disc

*Note:* Website: https://www.ors.org/spine‐travelfellowship/.

## 5 The Role of *JOR Spine*: A Journal Dedicated to Spine Research

A survey conducted by the ORS Spine Section together with the clinically focused society ISSLS to understand the publication trends of ORS Spine Section and ISSLS members [10] revealed that existing spine journals were not fully meeting the diverse needs of the spine community. This highlighted a clear opportunity to consolidate the field's most significant basic and translational discoveries in a dedicated, open‐access spine journal. As a result, the ORS Spine Section leadership developed the *JOR Spine*, led by three distinguished scientists representing the European (Mauro Alini), Asian (Daisuke Sakai), and North American (Robert L. Mauck) spine communities who served as the founding Editors‐in‐Chief (EICs) of this globally oriented, open‐access, spine‐specific journal.

As the ORS Spine Section celebrates its 10th anniversary, its growth has paralleled the emergence of *JOR Spine* as a dedicated platform for high‐quality pre‐clinical science and translational spine research for orthopedics. The journal has provided a focused venue for foundational and translational discoveries spanning aging, degeneration, biologic therapies, biomaterials, tissue engineering, biomechanics, bioreactors, culture systems, deformity and artificial intelligence. The journal's publications include scientific research articles as well as contributions highlighting initiatives by ORS members aimed at advancing science and addressing topics of importance to the scientific community. These works are presented as research articles [8, 9, 11, 12, 13, 14, 15, 16, 17, 18, 19], editorials [20, 21, 22, 23, 24], ORS Spine Section initiatives [7, 25, 26, 27, 28, 29, 30, 31], and memorial pieces [32].

**FIGURE 4:**
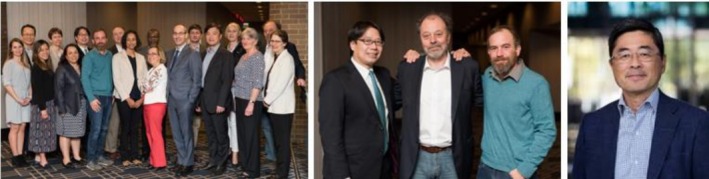
(left) The JOR Spine Advisory Board; (center) the inaugural *JOR Spine* Co‐Editors‐in‐Chief: Daisuke Sakai, Mauro Alini, Robert L. Mauck (2018–2025); (right) the current *JOR Spine* Editor‐in‐Chief: Keita Ito.

Publications of the journal have grown from 33 (46 submissions) in 2018 to 126 (525 submissions) in 2025, with a current acceptance rate of 24%. In 2025, *JOR Spine's* impact factor was 3.9, noting it as one of the most respected spine journals in the field. The tenure of the founding Co‐EICs (Mauro Alini, Robert L. Mauck, and Daisuke Sakai) ended in 2025; following this, Keita Ito was installed as the next EIC, with Cheryle Seguin, Fabio Galbusera, and Ashish Diwan as Associate Editors. Over the past decade, *JOR Spine* has strengthened interdisciplinary collaboration, increased the visibility of spine research, and supported the community through various initiatives, such as the *JOR Spine Early Career Award*. Together, the Spine Section and journal share a common mission: advancing impactful science that improves patient outcomes and fosters a strong global spine research community (Figure 4).

## 6 Lessons From a Decade of Leadership

**FIGURE 5:**
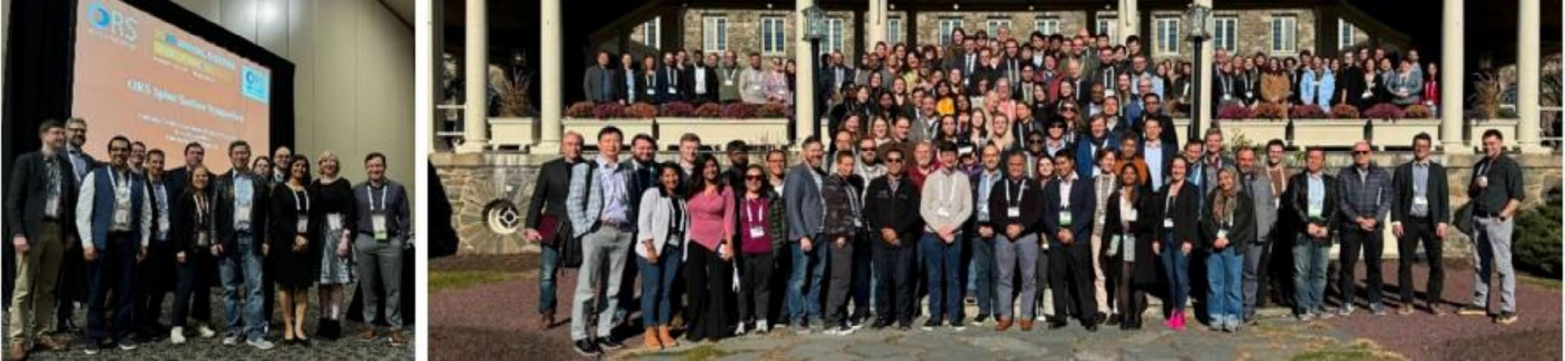
(left) The ORS Spine Section leadership group photo at the ORS 2025 Annual Meeting in Phoenix, Arizona, USA and (right) ORS spine community attendees at the ORS/PSRS International Spine Research Symposium in 2024 [22] at the Skytop Lodge in Skytop, Pennsylvania, USA.

Ten years of progress have highlighted several enduring principles for the ORS Spine Section. First, the scientific community drives impactful research, as breakthrough science thrives in collaborative and supportive environments. Second, leadership functions as stewards, with each Chair maintaining continuity while fostering innovation. Third, sustained investment in trainees is essential, as structured mentorship and leadership opportunities ensure the long‐term vitality of the field, with trainees receiving personalized mentorship, aiding them to thrive in careers in academia and industry. Finally, interdisciplinary collaboration accelerates translation, with the integration of biology, engineering, and clinical insight enabling meaningful scientific and clinical advances. These lessons have shaped the Section's growth over the past decade and will remain foundational as it moves forward (Figure 5).

## Author Contributions

**Neharika Bhadouria:** data curation, interpretation, writing original draft. **Dennis E. Anderson:** writing – review and editing. **Gabriela Graziani:** writing – review and editing, data curation. **Cheryle Séguin:** writing – review and editing. **Rita Kandel:** writing – review and editing. **Anthony Kirilusha:** writing – review and editing. **Ana V. Chee:** writing – review and editing. **Morgan B. Giers:** writing – review and editing. **Graciosa Q. Teixeira:** writing – review and editing. **Chitra L. Dahia:** writing – review and editing. **Aaron J. Fields:** writing – review and editing. **David J. Nuckley:** writing – review and editing. **Keita Ito:** writing – review and editing. **Derek H. Rosenzweig:** writing – review and editing. **Uruj Zehra:** writing – review and editing. **Fackson Mwale:** writing – review and editing. **Dmitriy Sheyn:** writing – review and editing. **Joshua Li:** writing – review and editing. **Nadeen O. Chahine:** writing – review and editing, data curation. **Robert L. Mauck:** writing – review and editing. **James C. Iatridis:** writing – review and editing. **Sibylle Grad:** writing – review and editing. **Christine L. Le Maitre:** writing – review and editing, data curation. **Makarand V. Risbud:** data curation, writing – review and editing. **Grace D. O'Connell:** writing – review and editing. **Judith A. Hoyland:** writing – review and editing. **Dino Samartzis:** conceptualization, supervision, interpretation, data curation, writing – review and editing. All other authors interpretation, data curation, and writing – review and editing.

## Funding

The authors have nothing to report.

## Conflicts of Interest

The authors declare no conflicts of interest.

## Data Availability

Data sharing not applicable to this article as no datasets were generated or analyzed during the current study.

## References

[1] M. V. Risbud , Z. R. Schoepflin , F. Mwale , et al., “Defining the Phenotype of Young Healthy Nucleus Pulposus Cells: Recommendations of the Spine Research Interest Group at the 2014 Annual ORS Meeting,” Journal of Orthopaedic Research 33, no. 3 (2015): 283–293, 10.1002/jor.22789. 25411088 PMC4399824

[2] J. C. Iatridis , J. Kang , R. Kandel , and M. V. Risbud , “New Horizons in Spine Research: Intervertebral Disc Repair and Regeneration,” Journal of Orthopaedic Research 35, no. 1 (2017): 5–7, 10.1002/jor.23499. 28114734 PMC5482231

[3] J. C. Iatridis , J. Kang , R. Kandel , and M. V. Risbud , “New Horizons in Spine Research: Disc Biology, Spine Biomechanics and Pathomechanisms of Back Pain,” Journal of Orthopaedic Research 34, no. 8 (2016): 1287–1288, 10.1002/jor.23375. 27571441 PMC5072778

[4] N. V. Vo , R. A. Hartman , P. R. Patil , et al., “Molecular Mechanisms of Biological Aging in Intervertebral Discs,” Journal of Orthopaedic Research 34, no. 8 (2016): 1289–1306, 10.1002/jor.23195. 26890203 PMC4988945

[5] L. S. Chakraborty , C. L. Le Maitre , N. O. Chahine , et al., “Impact of the COVID‐19 Pandemic on the Productivity and Career Prospects of Musculoskeletal Researchers,” Journal of Orthopaedic Research 42, no. 10 (2024): 2296–2306, 10.1002/jor.25866. 38678396

[6] J. T. Martin , D. Asimakopoulos , A. L. Hornung , et al., “Bullying, Harassment, and Discrimination of Musculoskeletal Researchers and the Impact of the COVID‐19 Pandemic: An International Study,” European Spine Journal 32, no. 6 (2023): 1861–1875, 10.1007/s00586-023-07684-7. 37014436 PMC10071222

[7] S. N. Tang , A. F. Bonilla , N. O. Chahine , et al., “Controversies in Spine Research: Organ Culture Versus In Vivo Models for Studies of the Intervertebral Disc,” JOR Spine 5 (2022): e1235, 10.1002/jsp2.1235. 36601369 PMC9799089

[8] D. Sakai , R. Leon , and M. Alini , “Advancing Spinal Research Beyond COVID‐19,” JOR Spine 6, no. 1 (2023): e1255, 10.1002/jsp2.1255. 36994460 PMC10041368

[9] M. T. Nolte , G. K. Harada , P. K. Louie , et al., “COVID ‐19: Current and Future Challenges in Spine Care and Education—A Worldwide Study,” JOR Spine 3, no. 4 (2020): e1122, 10.1002/jsp2.1122. 33392457 PMC7770197

[10] J. T. Martin , S. E. Gullbrand , A. J. Fields , et al., “Publication Trends in Spine Research From 2007 to 2016: Comparison of the Orthopaedic Research Society Spine Section and the International Society for the Study of the Lumbar Spine,” JOR Spine 1, no. 1 (2018): e1006, 10.1002/jsp2.1006. 29770804 PMC5944392

[11] M. D. Humphreys , L. Ward , S. M. Richardson , and J. A. Hoyland , “An Optimized Culture System for Notochordal Cell Expansion With Retention of Phenotype,” JOR Spine 1, no. 3 (2018): e1028, 10.1002/jsp2.1028. 31463448 PMC6686815

[12] J. J. Costi , E. H. Ledet , and G. D. O'Connell , “Spine Biomechanical Testing Methodologies: The Controversy of Consensus vs Scientific Evidence,” JOR Spine 4, no. 1 (2021): e1138, 10.1002/jsp2.1138. 33778410 PMC7984003

[13] C. L. Dahia , J. C. Iatridis , and M. V. Risbud , “New Horizons in Spine Research: Disc Biology, Tissue Engineering, Biomechanics, Translational, and Clinical Research,” JOR Spine 1, no. 3 (2018): e1032, 10.1002/jsp2.1032. 30687810 PMC6345533

[14] C. A. Séguin , D. Chan , C. L. Dahia , and Z. Gazit , “Latest Advances in Intervertebral Disc Development and Progenitor Cells,” JOR Spine 1, no. 3 (2018): e1030, 10.1002/jsp2.1030. 30687811 PMC6338208

[15] C. Jain , J. J. Huang , Y. Lee , et al., “Animal Models of Disc Degeneration Using Puncture Injury: A 20 Year Perspective,” JOR Spine 8, no. 3 (2025): e70093, 10.1002/jsp2.70093. 40727550 PMC12301940

[16] E. G. Buettmann , C. Chlebek , C. A. Lockard , S. W. Clayton , K. J. Lewis , and K. H. Collins , “Post or Perish? Social Media Strategies for Disseminating Orthopedic Research,” Journal of Orthopaedic Research 41, no. 8 (2023): 1643–1652, 10.1002/jor.25588. 37163368 PMC10524931

[17] A. M. Hollenberg , D. N. Bernstein , M. Beltejar , T. Terry , and A. Mesfin , “Publication Rate of Podium Presentations From the Orthopaedic Research Society Annual Meeting,” Journal of Orthopaedic Research 37, no. 2 (2019): 288–292, 10.1002/jor.24144. 30255536

[18] T. Alliston , K. C. Foucher , B. Frederick , et al., “The Importance of Diversity, Equity, and Inclusion in Orthopedic Research,” Journal of Orthopaedic Research 38, no. 8 (2020): 1661–1665, 10.1002/jor.24685. 32267012

[19] N. Bhadouria , J. Tiao , A. Baburova , et al., “Injury Induces More Severe Biomechanical Changes in Middle‐Aged and Geriatric Lumbar Spines in a Mouse Ex Vivo Model,” JOR Spine 8, no. 4 (2025): e70127, 10.1002/jsp2.70127. 41116840 PMC12535818

[20] R. Mauck , D. Sakai , and M. Alini , “JOR Spine: A (First) Year in Review,” JOR Spine 1, no. 4 (2018): e1041, 10.1002/jsp2.1041. 31463456 PMC6686822

[21] L. J. Smith , J. C. Iatridis , and C. L. Dahia , “Advancing Basic and Preclinical Spine Research: Highlights From the ORS PSRS 5th International Spine Research Symposium,” JOR Spine 3, no. 4 (2020): e1134, 10.1002/jsp2.1134. 33392462 PMC7770190

[22] C. L. Dahia , L. J. Smith , M. V. Risbud , and B. Gantenbein , “Advancing Basic and Preclinical Spine Research: Highlights From the ORS PSRS 7th International Spine Research Symposium,” JOR Spine 9, no. 1 (2026): e70161, 10.1002/jsp2.70161. 41728056 PMC12918395

[23] M. Alini , R. Mauck , and D. Sakai , “Welcome to JOR Spine!,” JOR Spine 1, no. 1 (2018): e1009, 10.1002/jsp2.1009. 31463439 PMC6686821

[24] C. L. Dahia and C. L. Le Maitre , “Improving Reproducibility in Spine Research,” JOR Spine 3, no. 3 (2020): e1127, 10.1002/jsp2.1127. 33015583 PMC7524211

[25] A. Lai , J. Gansau , S. E. Gullbrand , et al., “Development of a Standardized Histopathology Scoring System for Intervertebral Disc Degeneration in Rat Models: An Initiative of the ORS Spine Section,” JOR Spine 4, no. 2 (2021): e1150, 10.1002/jsp2.1150. 34337335 PMC8313153

[26] C. L. Dahia , J. B. Engiles , S. E. Gullbrand , et al., “A Perspective on the ORS Spine Section Initiative to Develop a Multi‐Species JOR Spine Histopathology Series,” JOR Spine 4, no. 2 (2021): e1165, 10.1002/jsp2.1165. 34337339 PMC8313167

[27] C. L. Le Maitre , C. L. Dahia , M. Giers , et al., “Development of a Standardized Histopathology Scoring System for Human Intervertebral Disc Degeneration: An Orthopaedic Research Society Spine Section Initiative,” JOR Spine 4, no. 2 (2021): e1167, 10.1002/jsp2.1167. 34337340 PMC8313169

[28] R. L. Mauck , M. Alini , and D. Sakai , “A Common Language for Evaluating Disc Degeneration and Regeneration: A JOR Spine/ORS Spine Section Initiative,” JOR Spine 2 (2019): e1056, 10.1002/jsp2.1056. 31463466 PMC6686790

[29] I. P. Melgoza , S. S. Chenna , S. Tessier , et al., “Development of a Standardized Histopathology Scoring System Using Machine Learning Algorithms for Intervertebral Disc Degeneration in the Mouse Model—An ORS Spine Section Initiative,” JOR Spine 4, no. 2 (2021): e1164, 10.1002/jsp2.1164. 34337338 PMC8313179

[30] N. N. Lee , E. Salzer , F. C. Bach , et al., “A Comprehensive Tool Box for Large Animal Studies of Intervertebral Disc Degeneration,” JOR Spine 4, no. 2 (2021): e1162, 10.1002/jsp2.1162. 34337336 PMC8313180

[31] L. J. Smith , L. Silverman , D. Sakai , et al., “Advancing Cell Therapies for Intervertebral Disc Regeneration From the Lab to the Clinic: Recommendations of the ORS Spine Section,” JOR Spine 1, no. 4 (2018): e1036, 10.1002/jsp2.1036. 30895277 PMC6419951

[32] M. Alini , R. Mauck , and D. Sakai , “In Memory of Peter Roughly and John Mort: Time for a “Biochemical” Reflection,” JOR Spine 2, no. 3 (2019): e1062, 10.1002/jsp2.1062. 31572979 PMC6764788

